# Post-angiography Retention of the Contrast Agent in the Left Atrial Appendage Is Associated With Risk of Cardioembolic Stroke in Patients With Atrial Fibrillation: A Retrospective Study

**DOI:** 10.3389/fcvm.2021.753949

**Published:** 2021-10-28

**Authors:** Ping Fang, Youquan Wei, Jinfeng Wang, Xianghai Wang, Hao Yang

**Affiliations:** Department of Cardiology, The First Affiliated Hospital (Yijishan Hospital) of Wannan Medical College, Wuhu, China

**Keywords:** atrial fibrillation, CHA_2_DS_2_-VASc score, left atrial appendage, angiography, cardioembolic stroke

## Abstract

**Background:** Atrial fibrillation (AF) represents an important risk factor for cardioembolic stroke, and most atrial thrombi originate from the left atrial appendage (LAA). Although the CHA_2_DS_2_-VASc score is widely used to estimate the risk of cardioembolic stroke in AF patients, yet greatly affected by many factors. This study was undertaken to determine the association between contrast agent retention in LAA after LAA angiography and risks of cardioembolic stroke in patients with AF.

**Methods:** This is a retrospective study. The demographic and clinical data of AF patients undergone left atrial appendage occlusion (LAAO) with or without catheter radiofrequency ablation were retrospectively analyzed. The patients were classified into either stroke or non-stroke group by the history with cardioembolic stroke or transient ischemic attack (TIA).

**Results:** Sixty-two consecutive patients undergone LAAO were finally included, in whom 31 AF patients had a history of cardioembolic stroke or TIA (one TIA), and significantly higher CHA_2_DS_2_-VASc score (4.2 ± 1.4 vs. 3.3 ± 1.3; *P* = 0.006) as well as incidence of contrast agent retention in LAA (*n* = 20 vs. *n* = 7; *P* = 0.001) compared to the patients in non-stroke group. In addition, the relative proportion of distinctive morphological types of LAA was significantly different between groups (*P* < 0.001). Multivariate logistic regression analysis showed that higher CHA_2_DS_2_-VASc scores (*OR* = 1.7, 95% *CI*: 1.0–3.0, *P* = 0.046) and LAA contrast agent retention (*OR* = 5.1, 95% *CI*: 1.1–23.9, *P* = 0.002) were associated with increased risks of cardioembolic stroke. The patients with Windsock type LAA (*OR* = 7.8, 95% *CI*: 1.1–57.2, *P* = 0.044) and Cauliflower LAA (*OR* = 20.2, 95% *CI*: 3.2–125.5, *P* = 0.001) were more prone to cardioembolic stroke compared to those with Chicken Wing type LAA.

**Conclusion:** Left atrial appendage contrast agent retention after LAA angiography is associated with the risks of cardioembolic stroke in patients with AF, and cardioembolic stroke is more seen in AF patients with Windsock or Cauliflower type LAA.

## Introduction

Atrial fibrillation (AF) represents a supraventricular arrhythmia, and commonly occurs in the aged population. This condition often progresses to impaired atrial contraction, reduced atrial emptying that can lead to blood stasis, thrombogenesis, and thromboembolism. Previous studies have shown that most atrial thrombi can arise from the left atrial appendage (LAA) (1–3). Also, AF can increase potential cardioembolic stroke by five-folds (4), and AF-related cardioembolic stroke is more fatal compared to the non-cardioembolic counterparts (5). Currently, CHA_2_DS_2_-VASc score is widely recommended to estimating the incidence of ischemic stroke in AF patients. According to the international guidelines, anticoagulation therapy should be considered for men with scores >1 and women with scores over 2 (6, 7). However, the CHA_2_DS_2_-VASc score appears incompetent to translate other risk factors responsible for stroke, such as left atrial diameter, alcohol abuse, chronic kidney disease (CKD), obstructive sleep apnea (OSA), duration of AF episodes, etc. Some investigations have confirmed that the morphology, volume and flow velocity of LAA are also associated with the risks of cardioembolic stroke in patients with AF (8–16). In other words, no definite consensus is reached at present for evaluating the risk of cardioembolic stroke in patients with AF.

Several clinical studies have shown that conventional anticoagulants such as warfarin or new oral anticoagulants (NOAC) can lower the risk of ischemic stroke in AF patients (17, 18). However, nearly 40% of the AF patients at risk of developing cardioembolic stroke do not receive anticoagulation therapy due to the concern of risk of internal bleeding, physician's decision, or other considerations (19), for which the differences are clear among countries in the proportion of patients with AF received anticoagulant therapy. Lip et al. reported that in Europe 80.5% AF patients with CHA_2_DS_2_-VASc score ≥1 received anticoagulant therapy (20), yet Xiang et al. reported that only 29.1% hospitalized AF patients with CHA_2_DS_2_-VASc score ≥1 underwent anticoagulant therapy in China (21). The proportion of outpatient patients with AF treated with anticoagulant therapy may be lower, so the incidence of ischemic stroke in China remains high. Although the proportion of patients receiving anticoagulant therapy tends to grow in recent years, a considerable number of patients with AF failed to receive anticoagulant medication. For these patients, left atrial appendage occlusion (LAAO) has emerged as a potential alternative to prevent ischemic stroke. In our clinical practice, we have observed that the contrast agent was retained after LAA angiography in the LAAO procedures in some patients. Nevertheless, few studies so far are available to demonstrate the correlation between contrast agent retention and the risk of cardioembolic stroke in patients with AF. The aim of this study therefore was to determine whether the retention of contrast agent after LAA angiography has an association with the risk of cardioembolic stroke in the AF patients.

## Methods

### Study Design

**Figure 1:**
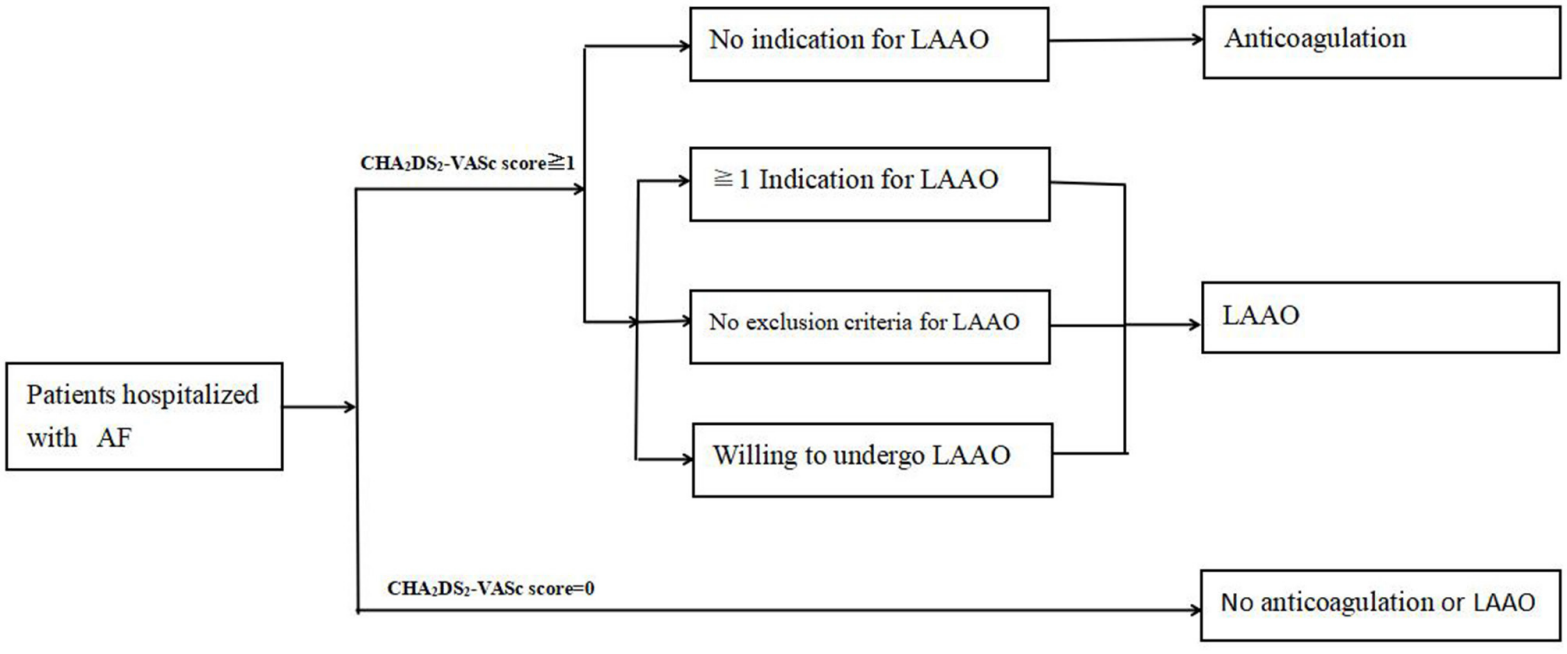
Flow chart of patient selection.

This is a retrospective study performed in single center. The clinical and demographic data were obtained from 65 AF patients undergone LAAO with or without radiofrequency catheter ablation (RFCA) at the First Affiliated Hospital of Wannan Medical College between August 1, 2019 and June 30, 2021. The 65 patients were the first to undergo LAAO in our center. The demographic data included age, gender, history of smoking, and alcoholism, and clinical data consisted of diameter of left atrium (LA), left ventricular ejection fraction (LVEF), AF type (persistent or paroxysmal), AF duration, antithrombotic therapy, history of cardioembolic stroke/transient ischemic attack (TIA), pre-and post-stroke CHA_2_DS_2_-VASc scores, heart failure, hypertension, diabetes, the estimated glomerular filtration rate (eGFR), body mass index (BMI), OSA, and intra-procedural images of LAA angiography. Patients with incomplete medical records were excluded. The study was approved by Institutional Review Board (IRB) of the First Affiliated Hospital of Wannan Medical College. Informed consent was obtained from each patient. Left atrial appendage occlusion is currently recommended as an alternative to anticoagulant therapy in patients with AF because of anticoagulant contraindications (22, 23). In view of the higher incidence of cardioembolic stroke in China due to lower proportion of anticoagulant therapy in AF patients, thus we attempted to bend the recognized recommendations for indications of LAAO in order to include more AF patients. These indications were composed of: (1) incapable of long-term anticoagulation from cerebrovascular or gastrointestinal bleeding history, poor compliance, or increased bleeding tendency; (2) history of cardioembolic stroke/TIA; (3) CHA_2_DS_2_-VASc scores ≥5; and (4) non-compliance with oral anticoagulant medication and request for non-pharmacological treatment. The exclusion criteria for LAAO in current study complied with the clinical study conducted in China (24), which included: atrial thrombus; LVEF <30%; symptomatic carotid disease; acute myocardial infarction or unstable angina; prior stroke or TIA within 30 days; acute infective endocarditis; pregnancy; prosthetic valve; atrial septal repair or closure history. The inclusion was illustrated in Figure 1.

### Diagnosis of Cardioembolic Stroke/TIA

All patients with a history of cardioembolic stroke/TIA in this retrospective study were diagnosed by experienced neurologists in our hospital. The diagnosis of cardioembolic stroke/TIA complied with the criteria of Modified TOAST classification of subtypes of ischemic stroke (25) (Supplementary Table 1). Patients with any of the following conditions, including cardioembolic stroke such as recent myocardial infarction, patent foramen ovale, valvular artificial prosthesis, and infective endocarditis, were ruled out (26).

### Antithrombotic Treatment for the Patients

All participants were treated with anticoagulant regimen for at least 1 month before LAAO procedure with or without RFCA, and combined anticoagulant and antiplatelet therapy, if which was required for other conditions. Heparin was administered intravenously to achieve an activated clotting time between 250 and 300 s during the procedure. Aspirin combined with clopidogrel antiplatelet was applied to each patient for 6 months after LAAO procedure, followed by single aspirin regimen. In this study, the pre-procedural, intra-procedural, and post-procedural antithrombotic treatments followed the protocols described in two prospective clinical studies on percutaneous LAmbre device implantation (27, 28).

### LAA Angiography

**Figure 2:**
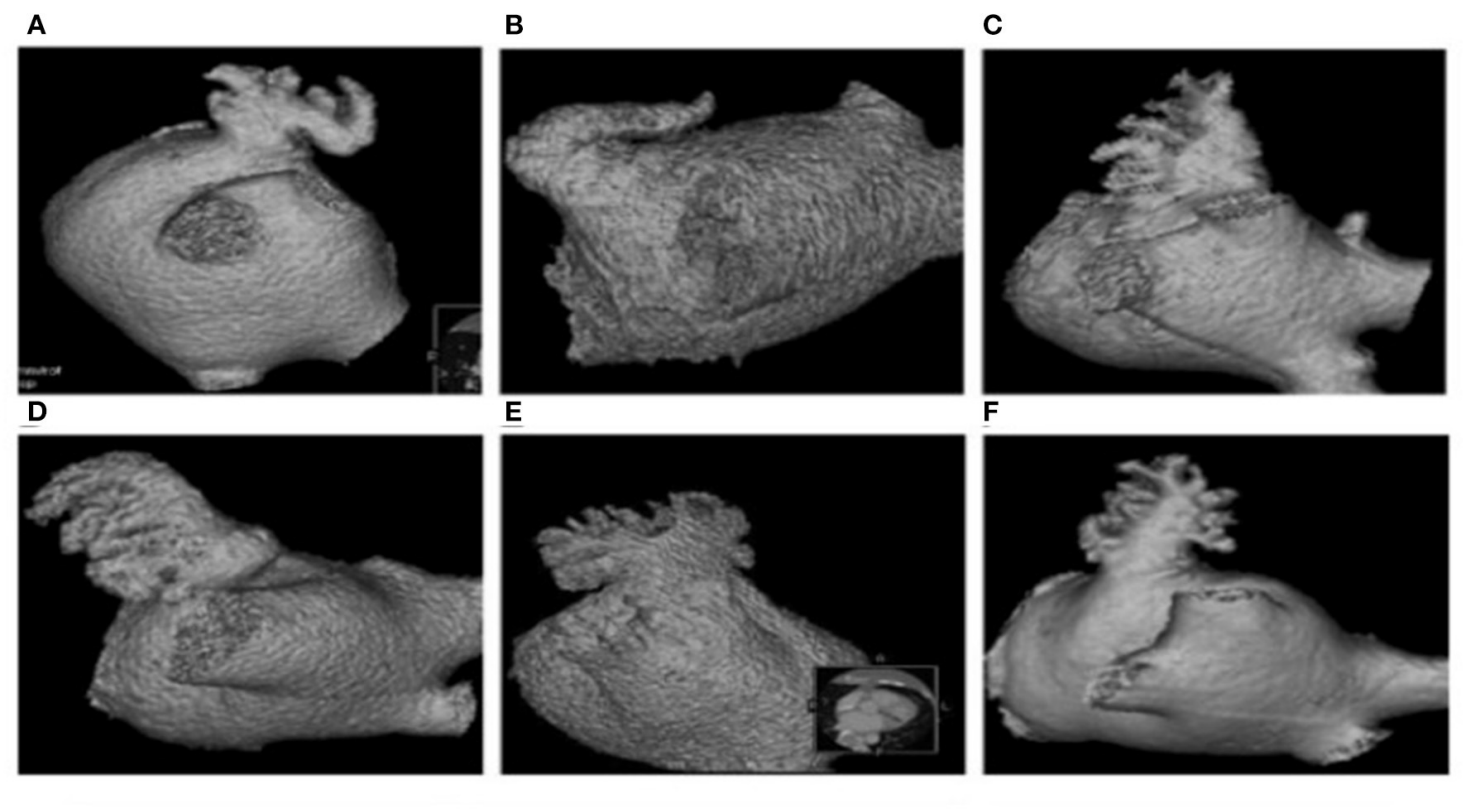
Morphological types of LAA. Chicken Wing **(A,B)**, Windsock **(C,D)**, cauliflower **(E)**, and cactus **(F)**.

Atrial fibrillation was initially ablated in the event that a patient was to be treated with LAAO plus AF ablation. External electrical cardioversion of AF was executed in the patients with failed restoration of the sinus rhythm upon completion of AF ablation. Left atrial appendage occlusion was performed using LAmbre device (Lifetech Scientific Co., Shenzhen, China) after puncturing the atrial septum. A pig tail tube was delivered to the LAA through LAmbre occluding sheath guided by X-ray fluoroscopy. The contrast agent was then injected into the LAA at RAO 30°/CUAD 20° through the tube and LAmbre occluding sheath, and the images were simultaneously taken. The orifice size and the lobe of LAA were measured. In addition, the LAA was classified into the Chicken Wing, Windsock, Cactus, and Cauliflower morphological types as shown in Figure 2 by Wang et al. (29), based on which LAA angiography was implemented in our study. The morphology of the LAA was independently determined by two specialists. Incidence of contrast agent retention after LAA angiography was maintained in all patients. In current work, contrast agent retention in the LAA was defined as the presence of the dye within 10 cardiac cycles following LAA angiography.

### Statistics

All statistical analyses were performed using softward SPSS version 22.0 (Inc. Chicago, IL). Continuous variables were expressed as mean ± SD for normally distributed data, and compared using two-sample *t*-test or paired *t*-test. Categorical variables were presented as numbers or percentages and compared using the χ^2^-test or Fisher's exact test. Multivariate logistical regression analysis was done to identify risk factors for cardioembolic stroke and the odds ratio (OR), and 95% confidence intervals (95% CI) were calculated. *P*-value < 0.05 was considered statistically significant.

## Results

**Figure 3:**
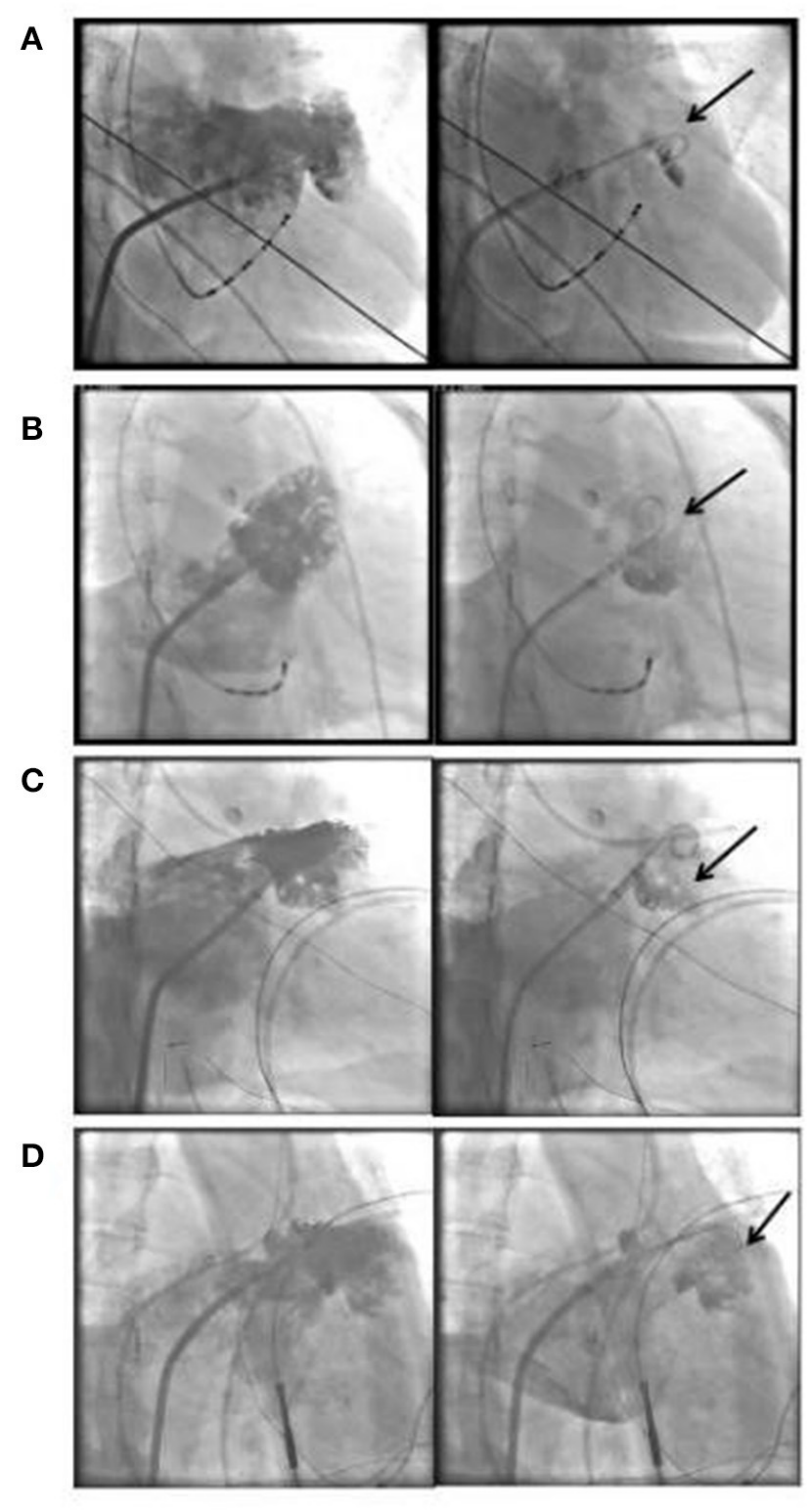
Illustration of LAA angiography image and contrast agent retention. Chicken Wing type LAA and contrast agent retention **(A)**, Cauliflower type LAA and contrast agent retention **(B–D)**, the arrows indicate contrast agent retention. LAA, left atrial appendage.

A total of 65 consecutive patients underwent LAAO with or without RFCA at the First Affiliated Hospital of Wannan Medical College during August 1, 2019 and June 30, 2021. Three patients with incomplete medical records were excluded. Finally, 62 consecutive patients were included in current investigation, of whom 30 had a history of cardioembolic stroke and one had TIA 1 month prior to admission. All patients received at least 1 month of anticoagulant therapy before LAAO intervention. No patients in the stroke group received antiplatelet therapy, and only one patient in non-stroke group underwent monotherapy with antiplatelet agent plus anticoagulant attributable to the percutaneous coronary intervention (PCI) procedure within 6 months. Fourteen patients in the stroke group and 16 in the non-Stroke group, respectively, underwent both ablation of AF and LAAO. Although patients in the stroke group had significantly higher CHA_2_DS_2_-VASc scores compared to those in the non-stroke group (4.2 ± 1.4 vs. 3.3 ± 1.3; *P* = 0.006), the former had lower pre-stroke scores (2.2 ± 1.4 vs. 3.3 ± 1.3; *P* = 0.005). No significant differences were observed regarding remaining baseline characteristics (*P* > 0.05). The demographic and clinical characteristics of all patients were summarized in Table 1. Left atrial appendage angiography showed that the type of Cauliflower morphology was most common (*n* = 20, 64%) in the stroke group, whereas the Chicken Wing type was more seen in the non-stroke group (*n* = 19, 61%). The relative proportions of the different LAA morphological types were significantly different between the two groups (*P* < 0.001). Furthermore, the proportion of stroke patients with contrast agent retention was significantly higher than that of those in the non-stroke group (64 vs. 23%, *P* < 0.001). Fifteen patients in the non-stroke group, and 17 in the stroke group, respectively, had ongoing AF upon our observation of contrast retention. Left atrial appendage angiography findings were summarized in Table 2, and some of the imaging manifestation were shown in Figure 3.

**Table 1:** The demographic and clinical characteristics for all participants.

	**Non-stroke group** **(***n*** = 31)**	**Stroke group** **(***n*** = 31)**	***P**-* **value**
Age, y^*^	71.8 ± 9.5	68.1 ± 8.9	0.111
CHA_2_DS_2_-VASc score	3.3 ± 1.3	4.2 ± 1.4	0.006
pre-stroke score	3.3 ± 1.3	2.3 ± 1.4	0.005
Gender, female, n^*^ (%)	14 (45%)	12 (39%)	0.607
BMI, kg/m^2^	24.8 ± 3.2	23.2 ± 3.1	0.050
Smoking, *n* (%)	8 (25%)	7 (23%)	0.767
Alcoholism, *n* (%)	1 (3%)	1 (3%)	1.000
AF type			0.354
Persistent AF, *n* (%)	30 (97%)	27 (90%)	
Paroxysmal AF, *n* (%)	1 (3%)	4 (10%)	
AF duration, months	53.7 ± 33.2	47.7 ± 34.2	0.482
OSA, *n* (%)	5 (16%)	6 (19%)	0.740
Heart failure, *n* (%)	12 (39%)	9 (29%)	0.421
Diabetes, *n* (%)	3 (10%)	4 (13%)	1.000
Hypertension, *n* (%)	13 (42%)	11 (35%)	0.602
eGFR, ml/min^*^	83.1 ± 17.7	83.9 ± 23.6	0.877
TTE measures
LA diameter (LAD), mm^*^	47.5 ± 5.2	46.4 ± 5.7	0.448
LVEF	61.4 ± 9.4	62.8 ± 4.2	0.436
Anticoagulant agent			0.755
Warfarin, *n* (%)	7 (33.3%)	6 (33.8%)	
NOAC, *n* (%)	24 (38.4%)	25 (40.8%)	
Antiplatelet agent, *n* (%)	1 (3%)	0	–
LAAO+AF ablation, *n* (%)	16 (51.6%)	14 (45.2%)	0.611

*Pre-stroke score, CHA_2_S_2_D-VASc score before stroke or TIA; BMI, body mass index; OSA, obstructive sleep apnea; eGFR, estimated glomerular filtration rate; TTE, transthoracic echocardiography; LA, center atrial; LVEF, center ventricular ejection fraction; NOAC, new oral anticoagulants*;

* *y, year; n, number; min, minute; mm, millimeter*.

**Table 2:** LAA angiography data.

	**Non-stroke group**	**Stroke group**	***P*** **-value**
	**(***n*** = 31)**	**(***n*** = 31)**	
Morphology of LAA			< 0.001
Chicken Wing, n^*^ (%)	19 (61%)	3 (10%)	
WindSock, *n* (%)	4 (13%)	4 (13%)	
Cactus, *n* (%)	5 (16%)	4 (13%)	
Cauliflower, *n* (%)	3 (10%)	20 (64%)	
LAA contrast agent retention *n* (%)	7 (23%)	20 (64%)	0.001

*LAA, Left atrial appendage*.

* *n, number*.

The univariate analysis found that morphology of LAA, CHA_2_DS_2_-VASc score, and LAA contrast agent retention were statistically significant between the two groups, therefore the three variables were included in the multivariable logistic regression analysis.

Multivariable logistic regression analysis showed that higher CHA_2_DS_2_-VASc score (*OR* = 1.7, 95% *CI*: 1.0–3.0, *P* = 0.046) and LAA contrast agent retention (*OR* = 5.1, 95% *CI*: 1.1–23.9, *P* = 0.002) significantly increased the risk of cardioembolic stroke. The patients with Windsock type LAA (*OR* = 7.8, 95% *CI*: 1.1–57.2, *P* = 0.044) and Cauliflower type LAA (*OR* = 20.2, 95% *CI*: 3.2–125.5, *P* = 0.001) were more likely to develop cardioembolic stroke compared to those with Chicken Wing type LAA (Table 3).

**Table 3:** Multivariable logistic regression analysis of factors associated with cardioembolic stroke.

**Variable**	**OR**	**95% CI**	***P*** **-value**
CHA_2_DS_2_-VASc score			0.046
CHA_2_DS_2_-VASc score = 1	1	–	–
CHA_2_DS_2_-VASc score > 1	1.7	1.0–3.0	
LAA type			0.010
Chicken Wing	1	–	–
WindSock	7.8	1.1–57.2	0.044
Cactus	3.0	0.4–22.2	0.277
Cauliflower	20.2	3.2–125.5	0.001
Contrast agent retention in LAA			0.002
No	1	–	–
Yes	5.1	1.1–23.9	

*CI, confidence interval; OR, odds ratio; LAA, left atrial appendage*.

## Discussion

Atrial fibrillation is the most common supraventricular arrhythmia, affecting an estimated 33.5 million victims worldwide (30). This condition can result in impaired atrial contraction, reduced atrial emptying, blood stasis, thrombogenesis, and cardioembolic stroke. The LAA is a finger-like projection extending from the LA, and regulating atrial pressure and endocrine function (31). Given the unique shape, LAA is the source of thromboembolism in more than 90% of the patients with non-valvular AF presenting with cardioembolic stroke (32). Stroke is currently ranked the fifth leading cause of death in the United States (33), and AF increases the risks of stroke by five-folds. Furthermore, cardioembolic stroke is more fatal than non-cardioembolic stroke (5). Patients with AF often exhibit atrial fibrosis, atrial dilatation, myofibrillar, and endothelial dysfunction (34, 35), as well as hemodynamic changes that result in thrombosis and increase the risk of stroke. Consequently, the pathophysiological changes aforementioned may lead to LAA thrombosis, even in the absence of AF (36, 37). CHA_2_DS_2_-VASc score is widely used to estimate the risk of cardioembolic stroke in AF patients. In our observations, we found that patients in stroke group had significantly higher CHA_2_DS_2_-VASc scores, whereas the pre-stroke scores were lower in this group compared to the non-stroke counterparts. One possible reason is that the majority of the patients included in current study without a history of cardioembolic stroke, though having relatively higher CHA_2_DS_2_-VASc scores, yet they were not tolerated to long-term anticoagulant administration, thus LAAO should be considered as an option in clinical practice. Another possibility is that some patients in the stroke group did not have high pre-stroke score despite being at a higher risk. Accordingly, the CHA_2_DS_2_-VASc score is incompetent in some cases for accurately identifying AF patients at a high risk of cardioembolic stroke.

The proportion of patients with contrast agent retention in LAA was significantly greater in the stroke vs. non-stroke group, and multivariable logistic regression analysis further showed that both CHA_2_DS_2_-VASc score and LAA contrast agent retention were associated with a high risk of cardioembolic stroke. Retention of the contrast agent after LAA angiography means that the patients' blood is also prone to stasis in the LAA, which creates a condition for LAA thrombosis. Interestingly, the relative proportion of the different morphological types of LAA were significantly different between groups. While Chicken Wing type seemed predominant in patients in the non-stroke group, yet Cauliflower type was more common in those in the stroke group. This is just consistent with the findings reported by Adukauskaite et al. that significant correlation exists between WindSock or Cauliflower LAA type and risks of cardioembolic stroke in AF patients (38). In addition, there is evidence demonstrating that the LA wall thickness is a risk factor for cardioembolic and cryptogenic stroke (39). Lee et al. found that the Chicken Wing LAA was related to a lower incidence of cardioembolic stroke, which can be attributed to the small size of this morphological type that is capable of enhancing the velocity of blood flow (40). In this study we also found that the patients with Windsock or Cauliflower type LAA were more inclined to having cardioembolic stroke compared to those with Chicken Wing type LAA.

To summarize, contrast agent retention after LAA angiography is strongly associated with the risks of cardioembolic stroke development in patients with AF. Therefore, routine LAA angiography is recommended for individual patient undergoing AF ablation in order to supplement the CHA_2_DS_2_-VASc score for risk stratification of cardioembolic stroke. Besides, LAA angiography can be used to identify AF patients at a high risk of cardioembolic stroke even if in patients with lower CHA_2_DS_2_-VASc score.

## Limitations

Some limitations in current study consist of: (1) retrospective observation in single center with limited samples, which needs further verification on a larger, multi-center cohort; (2) hard ensuring the final diagnosis of cardioembolic stroke because this condition can arise from diverse sources, consequently misdiagnosis of cardioembolic stroke/TIA in AF patients might be inevitable, even in the hands of experienced neurologists; and (3) potentially biased LAA morphology definition because of it being subjectively interpreted via angiography instead of digitized modeling by computed tomography (CT) or magnetic resonance imaging (MRI).

## Conclusions

Post-angiography retention of the contrast agent in the LAA has an association with the risk of cardioembolic stroke in patients with AF. Atrial fibrillation patients with Windsock or Cauliflower type LAA are prone to developing cardioembolic stroke. Left atrial appendage angiography can be used to identify the degree of risks for AF patients with cardioembolic stroke regardless of the CHA_2_DS_2_-VASc score. For patients with LAA contrast retention following LAA angiography, LAAO or long-term anticoagulant therapy should be maintained even if the CHA_2_DS_2_-VASc score remains lower.

## Data Availability Statement

The original contributions presented in the study are included in the article/Supplementary Material, further inquiries can be directed to the corresponding author/s.

## Ethics Statement

The studies involving human participants were reviewed and approved by the Institutional Ethics Committee for Biomedical Research of the First Affiliated Hospital of Wannan Medical College. The patients/participants provided their written informed consent to participate in this study.

## Author Contributions

PF: drafted the manuscript. PF, YW, JW, XW, and HY: performed the procedures and collected the data. HY and PF: designed this research and revised the manuscript. All authors read and approved the final version of the manuscript.

## Conflict of Interest

The authors declare that the research was conducted in the absence of any commercial or financial relationships that could be construed as a potential conflict of interest.

## Publisher's Note

All claims expressed in this article are solely those of the authors and do not necessarily represent those of their affiliated organizations, or those of the publisher, the editors and the reviewers. Any product that may be evaluated in this article, or claim that may be made by its manufacturer, is not guaranteed or endorsed by the publisher.
