# Supplementary material for: Post-angiography Retention of the Contrast Agent in the Left Atrial Appendage Is Associated With Risk of Cardioembolic Stroke in Patients With Atrial Fibrillation: A Retrospective Study
Source: Front Cardiovasc Med. 2021 Oct 28;8:753949. doi: 10.3389/fcvm.2021.753949 (PMC8581209; doi:10.3389/fcvm.2021.753949)
Supplement: Supplementary file 1 [file Table_1.DOCX]

Table 1. Modified TOAST classification of subtypes of ischemic stroke

（This table was cited from Fure, B. et al[25]）

|  | Clinical signs | Radiological signs | Supplementary investigations |
| --- | --- | --- | --- |
| Large vessel disease | Signs of lesion in cortex (aphasia, apraxia, neglect), subcortex, cerebellum or brainstem | CT or MRI shows lesion >1.5 cm in cortex, subcortex, cerebellum or brainstem compatible with the symptoms. CT scan negative when performed shortly after start of the symptoms | Color duplex images of precerebral arteries shows stenosis ≧50% or occlusion in symptomatic ICA/CCA |
| Cardio-embolic disease | Signs of lesion in cortex (aphasia, apraxia, neglect), subcortex, cerebellum or brainstem | CT or MRI shows lesion >1.5 cm in cortex, subcortex, cerebellum or brainstem compatible with the symptoms. CT scan negative when performed shortly after start of the symptoms | ECG/TEE confirm high risk or medium high risk of cardio-embolic source* |
| Small vessel disease | Clinical signs of a lacunar syndrome (pure motor, pure sensory, sensorimotor, atactic hemiparesis or dysarthria-clumsy hand syndrome) | CT or MRI shows lacunar infarction (infarction <1.5 cm) compatible with the symptoms. CT scan negative when performed shortly after start of the symptoms | No large vessel disease or cardio-embolic disease identified in color duplex images of precerebral arteries or in ECG/TEE |

ICA, internal carotid artery; CCA, common carotid artery; TEE, transesophageal echocardiography. *Medium high risk of cardio-embolic source: mitral stenosis without atrial fibrillation, atrial septum aneurysm, patent foramen ovale, atrial flutter, nonbacterial thrombotic endocarditis, congestive heart failure, hypokinetic left ventricular segment, myocardial infarction (>4 weeks, <6 months). *High risk of cardio-embolic source: mechanical heart valve, mitral stenosis with atrial fibrillation, atrial fibrillation, left atrial thrombus, recent myocardial infarction (<4 weeks), left ventricular thrombus, dilated cardiomyopathy, akinetic left ventricular segment, atrial myxoma, infectious endocarditis.
